# Genetic characterization of the bat and human lineages of the common bed bug (*Cimex lectularius*) at a local scale

**DOI:** 10.1017/S0031182025000575

**Published:** 2025-04

**Authors:** Clara Castex, Antoine Perrin, Laura Clément, Pierre Perréaz, Jérôme Goudet, Philippe Christe

**Affiliations:** Department of Ecology and Evolution, University of Lausanne, Lausanne, Switzerland

**Keywords:** *Cimex lectularius*, genetic differentiation, host races, insecticide resistance, local scale

## Abstract

After its near eradication in the 1940s, the common bed bug (*Cimex lectularius*) experienced a global resurgence. Within a few years after, some populations displayed insecticide resistance. Two distinct lineages of bed bugs were identified, each associated with humans and bats, respectively. A strong genetic differentiation was identified between bugs from human and bat sites across Europe. This raises the question of whether the same pattern is found at a local scale. Moreover, because long-distance dispersal of bed bugs is essentially human-mediated, we investigated the spread of bed bugs within and among sites. Using mitochondrial (cytochrome oxidase unit I (COI) and 16S rRNA genes) and nuclear (10 microsatellite loci) markers, we compared the genetic composition of human- and bat-associated bed bugs from western Switzerland. We first conducted a median-joining analysis and compared it to European sequences to detect local-scale host-specific separation of haplotypes. We estimated levels of genetic diversity and structure between and within the two host-associated bed bugs. Our results reveal two genetic clusters associated with bats and humans and a strong structure among human sites (*F*_SC_ = 0·579). An analysis of knock-down insecticide resistance gene variants (V419L, L925I, I936F) shows that bed bugs infecting humans in western Switzerland carry insecticide resistance (99%) whereas bed bugs infecting bats do not (0%). Our results show that at the scale of western Switzerland, bed bugs are structured by host association, thus supporting the hypothesis of host specialization in the common bed bugs. Moreover, human-associated bugs might have settled from multiple colonization events and/or undergone bottlenecks.

## Graphical abstract

**Figure:**
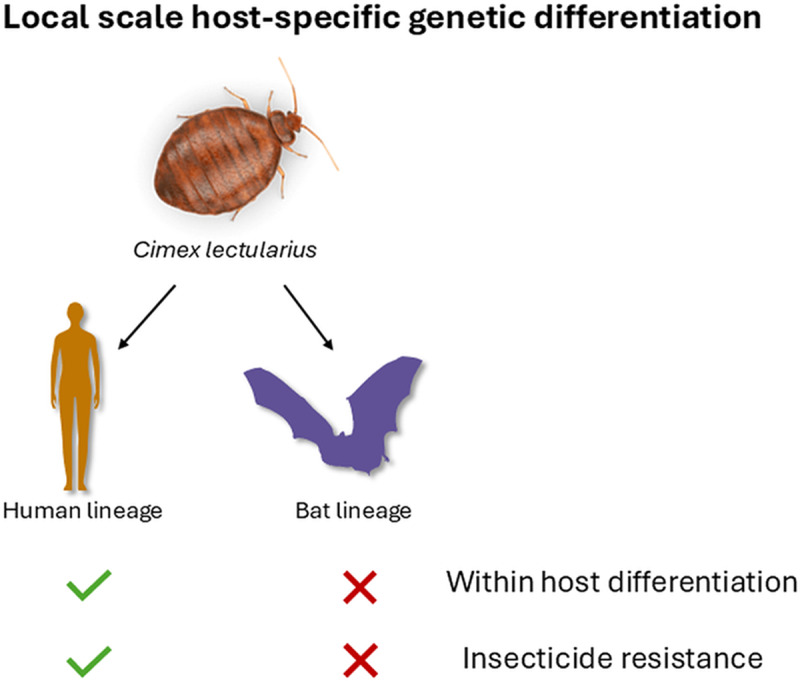

## Introduction

Parasites depend on their hosts to survive and reproduce and have developed a diverse array of life cycle complexities, host ranges and host switches for this reason (Poulin, 1995; Morand, 2015; Hay et al., 2020). One major driver of parasite diversification is host specialization, defined as the degree of association between a parasite and different host species (Dick and Patterson, 2007). This degree is variable between parasites with some generalist and more specialist species. Different measures have been described to assess host specialization such as the number of hosts exploited, the phylogenetic distance and the functional diversity of hosts (Poulin et al., 2011; Medina and Langmore, 2016). Such measures help understand how host specialization can lead to speciation or the formation of host races. Drès and Mallet (2002) defined host races as the genetic differentiation of lineages specialized on different hosts living in the same area. For example, the study of seabird ticks, *Ixodes uriae*, hosted by the black-legged kittiwake (*Rissa tridactyla*) and the Atlantic puffin (*Fratercula arctica*), has revealed a host-related diversification in the genetic structure of the ticks (Mccoy et al., 2003).

Host specialization is a phenomenon that is particularly visible in the *Cimicidae* bug family. *Cimicidae* are temporary ectoparasites that feed directly on their host and spend most of their time concealed within the host’s shelter (Usinger, 1966). These obligate blood-feeding parasites are widespread among different host species. Out of 22 genera of *Cimicidae*, 12 are bat-only and 9 are bird-only associated parasites (Roth et al., 2019). The *Cimex* genus contains 10 species and is recognized to be associated with several groups of hosts. Most of the *Cimex* species (e.g. *C. pipistrelli, C. adjunctus* or *C. pilosellus*) are specialized on bats, which are thought to be the ancestral host of the genus (Usinger, 1966). However, some of the *Cimex* species can be found on multiple hosts including bats, birds or even humans (e.g. *C. lectularius* and *C. hemipterus*). The common bed bug, *C. lectularius*, is predominant in temperate regions (Usinger, 1966; Zorrilla-Vaca et al., 2015).

Human-associated *C. lectularius* were almost eradicated between 1940 and 1950 in many areas due to intensive use of dichlorodiphenyltrichloroethane (DDT) insecticides (Barnes, 1946;Seong et al., 2010; Davies et al., 2012). However, nowadays bed bugs seem to spread again around the world (Levy Bencheton et al., 2011; Davies et al., 2012; Delaunay, 2012; Dang et al., 2015). One of the main reasons for this resurgence is an increase in insecticide tolerance (Romero et al., (2007); Zhu et al., 2010; Lewis et al., 2023). In addition, the intensification of human interactions, such as better access to long-distance transport (Delaunay, 2012), may contribute to this recent resurgence. It increases the risk of introducing resistant individuals into existing populations, making pest control methods less efficient. As a result, bed bug populations may continue to grow, leading to their wider spread as an urban pest species and further increasing their already significant public health and economic impact (Doggett et al., 2018; Hwang et al., 2018; Perron et al., 2018).

Ecological differences between bats and humans can result in different selective pressures, which may lead to host race formation in bed bugs. Common bed bugs *C. lectularius* found in human shelters and bat roosts were shown to be genetically differentiated (Balvín et al., 2012; Booth et al., 2015; Balvín and Booth, 2018). Additionally, based on the differences in feeding behaviour, survival and development, the two host-associated groups were defined as two distinct ecotypes according to Wawrocka and Bartonička (2013). This supports the morphological differentiation in sensory, feeding and dispersal organs (Balvín et al., 2012). However, except for Wawrocka et al. (2015), recent hybridization experiments did not find any evidence for post-mating barriers and reproductive isolation (Devries et al., 2020; Sasínková et al., 2023). Factors other than ecological could have shaped genetic differentiation between bat- and human-associated bed bugs. Indeed, as mentioned above, the intensive use of insecticide by humans has induced insecticide resistance around the world due to mutations in the voltage-gated sodium channel (Doggett et al., 2004; Zhu et al., 2010; Durand et al., 2012; Dang et al., 2015, 2017; Lilly et al., 2015; Palenchar et al., 2015; Raab et al., 2016; Cho et al., 2020; Vander Pan et al., 2020; Akhoundi et al., 2021; Lewis et al., 2023). If individuals were resistant, then we would expect this mutation to be present in bed bugs from human shelters and not in bugs from bat roosts that have not been exposed to insecticides, as pointed out by previous studies in Europe (Booth et al., 2015; Balvín and Booth, 2018).

The genetic differentiation of the two lineages of bed bugs was mostly investigated at large scale in Europe (Balvín et al., 2012; Booth et al., 2015; Balvín and Booth, 2018). This raises the question of whether the same pattern can occur between bat and human sites at the local scale. There are few records of local-scale genetic differentiation of bed bugs within hosts (Djouaher et al., (2024); Booth et al., 2012). Because the long-distance dispersal of the common bed bug between human sites is mediated by humans (Doggett et al., 2004; Reinhardt and Siva-Jothy, 2007; Delaunay, 2012), it would be interesting to explore if bed bugs are able to spread within and among sites at a more local scale.

Here, we performed genetic analyses to understand bed bug transmission between and within hosts at a local scale, in Western Switzerland. We examined genetic differentiation and gene flow both between and within bat- and human-associated bed bugs and compared it to previous results of differentiation across Europe (Booth et al., 2015). Genetic diversity and structure provided information on the extent of the differentiation of the two host-associated lineages of bed bugs at a local scale. We also investigated bed bug movements within hosts and disentangled whether gene flow occurred between human-associated sites and whether the same patterns were observed within bat roosts. Additionally, we tested insecticide resistance mutation as a potential genetic marker to differentiate bed bugs from bats and humans. We first estimated the proportion of the insecticide-tolerant bugs from the two hosts and then assigned the prevalence of each of those two tested mutations.

## Materials and methods

### Bed bugs collection and DNA extraction

We collected 417 *C. lectularius* (345 – on humans, 72 – on bats) from 30 human dwellings and 2 bat roosts (*Myotis myotis*), respectively (Figure S1, Table S1). The human-associated bugs were collected by various pest management professionals before any treatment was applied. All bugs were stored at −20°C in tubes filled with 90% ethanol. The bed bugs were rehydrated in distilled water for 30 min, and DNA was extracted using the DNeasy Blood and Tissue kit (Qiagen) following the manufacturer’s protocol.

### mtDNA analysis

A 658 bp fragment of the cytochrome oxidase unit I (COI) gene was amplified using the modified primers LepF (5ʹ-ATTCAACCAATCATAAAGATATTGG-3ʹ) and LepR (5ʹ-TAAACTTCTGGATGTCCAAAAAATCA-3ʹ) from Hajibabaei et al. (2006) according to Booth et al. (2015). In addition, a 382 bp fragment of the 16S rRNA gene was amplified using the primers LR-J-13007 (5ʹ-TTACGCTGTTATCCCTAA-3ʹ) (Kambhampati and Smith, 1995) and LR-N-13398 (5ʹ-CGCCTGTTTATCAAAAACAT-3ʹ) (Simon et al., 1994). Polymerase chain reaction (PCR) protocol from Rosli et al. (2011) was improved using 0·3 µm of primers, 3·0 mm of MgCl_2_ and 35 cycles. Amplifications were tested on a 1·5% agarose gel, and amplified fragments were sent for sequencing (Microsynth AG: Sanger method). Sequences were aligned in MEGA11 (Tamura et al., 2021). To compare the local haplotypes with the European ones, the reverse complement of 266 COI and the 16S sequences (214 human-associated bed bugs from 24 sites and 52 bat-associated bed bugs from 2 sites) were concatenated into a 948 bp sequence and aligned to 214 sequences of Booth et al. (2015), available on Dryad. A median-joining network (Bandelt et al., 1999) was built in PopArt v1.7 (Leigh and Bryant (2015)) using the default parameters. One hundred and forty-six individuals out of 417 were discarded because fragments failed to be sequenced, and 5 individuals were disregarded because of signal of heteroplasmy, frequent in the common bed bugs (Booth et al., 2015; Robison et al., 2015).

### Microsatellite analysis

An analysis of polymorphic sites was performed using 12 species-specific microsatellite loci (BB15B, BB21B, BB28B, BB29B, BB31B, BB38B, BB42B, Clec6, Clec11, Clec37, Clec48, Clec99) developed by Booth et al. (2012). The PCR were run according to Booth et al. (2012) with 0·3 µl of primer for each locus and an increase of 40 cycles. Taq amount was doubled for BB15B, BB21B, BB28B, BB29B and BB31B to increase the success of genotyping. PCR success was tested on 1·5% agarose gel. Sequencing was performed on a 3100 Genetic Analyzer (Applied Biosystems). Scoring of the various allele sizes was done by hand using GeneMapper 4.0 (Applied Biosystems). Micro-checker v2.2.3 (Van Oosterhout et al., 2004) was used to assess the presence of scoring errors, null alleles or large-allele dropout in each locus. Linkage disequilibrium was analysed between loci on 1000 permutations with the FSTAT software v2.9.4 (Table S2; Goudet, 2002). Because we lack a common panel, our microsatellite data could not be compared to the European microsatellite dataset from Booth et al. (2015). Among the 32 sites sampled in this study, 27 sites sampled in 2016 were genotyped to carry out the microsatellite analysis (Table S1). Out of the 12 tested loci, BB15B and BB28B showed null alleles and missing data, and thus 10 loci were kept in the analysis (BB21B, BB29B, BB31B, BB38B, BB42B, Clec6, Clec11, Clec37, Clec48 and Clec99). In total for the microsatellite analysis, 370 individuals from 25 human shelters (*N* = 311) and 2 bat roosts (*N* = 59) were analysed.

To assess the genetic diversity between and within sampling sites, we kept the 17 sites with 5 or more individuals. The following summary statistics were obtained with the ‘hierfstat’ R package 0.5.11 (Goudet et al., 2022; R Core Team, 2022): allelic richness per host (Ar), observed (Ho) and expected heterozygosity (He) and population inbreeding coefficient (*F*_IS_). The confidence intervals for *F*_IS_ per site were obtained by bootstrapping over loci with 1000 bootstraps. Paired Wilcoxon tests were performed to compare summary statistics between human and bat sites. Pairwise *F*_ST_ values were calculated to assess population structure between the host-associated sites using the method of Weir and Cockerham (1984). To test for population structure, we used the test.g function from the ‘hierfstat’ R package with 2720 randomizations to adjust for multiple testing. To estimate the genetic variance components between hosts and among sampling sites, a hierarchical analysis of variance was carried out using the varcomp.glob function of the ‘hierfstat’ R package and significance was tested with 1000 permutations. The 95% confidence interval of the variance components was also calculated over 1000 bootstraps using the boot.vc function of the same package.

To test for isolation by distance considering the structure of our dataset, the correlation between the pairwise *F*_ST_, the geographical distance between sites and two geographical structure matrices was estimated. The first structural matrix accounted for the fact that 10 of the 17 human sites were tightly clustered around Geneva and thus took a value of 1 if the pairwise sites both belonged to Geneva or did not belong to Geneva, and a value of 0 if one of the sites was from the Geneva cluster while the other was not. The second matrix accounted for bat roosts that were not sampled in the same places as human shelters (i.e. 1 for pairwise sites from the same host (bat or human) and 0 for pairwise sites from different hosts). Since the structural matrices were correlated with geographic distances (Table S3), and because of the non-independence of pairwise distances, a generalized least squares (GLS) model with a maximum-likelihood population effects correlation structure was conducted (MLPE, Clarke et al., 2002). The gls function from the ‘corMLPE’ package (Pope, 2024) was used for the entire dataset using only the structural matrices. Finally, the effect of geographical distances on genetic distances was tested on pairs of bed bugs from Geneva and on pairs of bed bugs from outside Geneva on human-associated bugs only, using a Mantel test.

Finally, using all 27 sites, we calculated the pairwise kinship between individuals with the beta.dosage function in ‘hierfstat’ (Goudet et al., 2022). Because of the high levels of relatedness (see Results section), a single specimen per site was randomly chosen, and the analysis was randomized 100 times. To examine the amount of substructure among the various bed bug sites collected on the two hosts, principal component analysis (PCA) between host-associated bed bugs was run using the indpca function implemented in ‘hierfstat’ (Goudet et al., 2022).

### Pyrethroid resistance: knock-down resistance mutation

Knock-down resistance (*kdr*) genotypes at the amino acids 419, 925 and 936 in the voltage-gated sodium channel were determined to assess the resistance of *C. lectularius* to pyrethroid insecticide. PCR was done to identify the three mutations in the voltage-gated sodium channel. The allele independent primers BBParaF1 (5′-AACCTGGATATACATGCCTTCAAGG-3′) and BBParaR1 (5′-TGATGGAGATTTTGCCACTGATG-3′) were used to amplify a fragment containing the amino acid 419, and BBParaF3 (5′-GGAATTGAAGCTGCCATGAAGTTG-3′) and BBParaR3 (5′-TGCCTATTCTGTCGAAAGCCTCAG-3′) were used to amplify the fragment containing the amino acids 925 and 936 (Zhu et al., 2010). PCR was run in total volume of 25 µl with the minimal amount of 20 ng DNA template, 0·3 µm of each primer, 0·5 U of Taq polymerase (Promega), 0·3 mm of dNTPs, 1× Taq buffer (Promega), and 0·5 mm MgCl_2_. PCR mix was completed to 25 µl with H_2_O MilliQ. The PCR conditions were 94°C for 3 min, followed by 35 cycles of 94°C for 30 s, 55°C for 30 s and 72°C for 1 min with a final extension step at 72°C for 10 min. Successfully, amplified products were sent for forward sequencing (Microsynth), with a successful sequencing of 342 individuals (with 55 and 287 individuals for bats and human-associated bed bugs, respectively). Sequences were then checked for the mutated base pairs for both locations known to be associated with *kdr* resistance: V419L (wild-type: valine = GTC; mutated: leucine = CTC), L925I (wild-type: leucine = CTT; mutated: isoleucine = ATT) and I936F (wild-type: isoleucine = ATT; mutated: phenylalanine = TTT) using MEGA11 software. Individuals were identified as heterozygotes for mutation where overlapping peaks occurred in the chromatograms (Dang et al., 2015). The annotation of Lewis et al. (2023) was used to designate all the *kdr* genotypes.

## Results

### mtDNA analysis: comparison with European haplotypes

**Figure 1.**
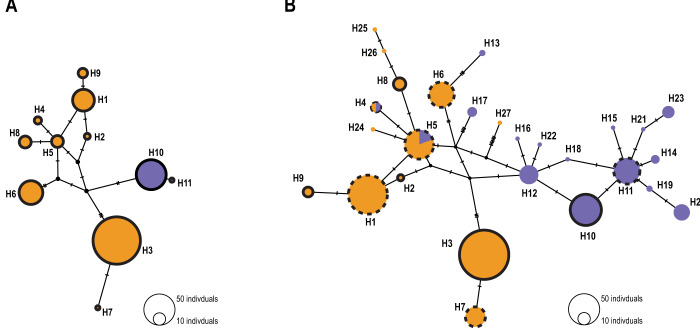
Median joining network based on the concatenated 16S and COI mitochondrial genes with (A) Swiss individuals only and (B) European individuals from Booth et al. (2015). Human-associated haplotypes are represented in orange, and bat-associated haplotypes are purple. The number of individuals sharing the same haplotype is highlighted by the size of the circles. The black dots represent ancestral or unsampled haplotypes. Traits denote the number of nucleotide differences between haplotypes. In (B), circles without outlines represent haplotypes from Booth et al. (2015), black circles highlight new haplotypes whereas black dotted circles represent shared haplotypes between the Swiss and the European dataset.

In the common bed bugs of western Switzerland only, the COI and 16S concatenated genes revealed 11 haplotypes. The haplotype median-joining network was consistent with a clear separation between the two hosts (Figure 1A). Among the 11 haplotypes, 9 were human-associated and 2 were bat-associated. No haplotype was shared between the two hosts (Figure S2; Table S4). In general, we observed the same pattern of host differentiation at the local scale and at the European scale. Indeed, with the addition of the European sequences, the analysis revealed a total of 27 haplotypes, 11 of which were associated with humans, 14 with bats and 2 with both host-associated sequences. Haplotypes were shared between sites. In total, 6 haplotypes were shared between the local and the European datasets (Figure 1B). Of these 6 haplotypes, 1 haplotype was strictly associated with bats, 3 haplotypes were associated with humans and 2 haplotypes shared both host-associated sequences. We identified 5 new haplotypes (H2, H3, H8, H9 and H10 in Figure 1B). Four of these were associated with humans, while H10 was exclusively associated with bats. (Figure S2).

### Microsatellite analysis

#### Genetic diversity

For all 10 microsatellite markers, our analyses with micro-checker (Van Oosterhout et al., 2004) found no evidence of scoring error, null allele presence or large allele dropout in the final dataset. After Bonferroni correction, there was no significant linkage disequilibrium. The gene diversity per population over loci was significantly different between hosts (paired Wilcoxon test: *V* = 50, *P* = 0·020), with bat-associated bugs more genetically diverse than human-associated lineages, despite a much lower sample size (Tables 1 and 2; Table S5). Similarly, we found higher allelic richness in the bat-associated bugs (paired Wilcoxon test: *V* = 50, *P* = 0·020). However, more private alleles were found in the human-associated bed bugs than the bat-associated ones (paired Wilcoxon test: *V* = 2·5, *P* = 0·019).

**Table 1.** Genetic diversity of the common bed bugs over loci

	Ar			Ho			He		
Locus	Overall	Bat	Human	Overall	Bat	Human	Overall	Bat	Human
BB21B	1·268	1·696	1·211	0·123	0·085	0·128	0·279	0·709	0·217
BB29B	1·305	1·522	1·276	0·141	0·000	0·160	0·349	0·534	0·317
BB31B	1·288	1·483	1·260	0·182	0·000	0·208	0·291	0·511	0·259
BB38B	1·360	1·605	1·327	0·320	0·633	0·278	0·367	0·604	0·335
BB42B	1·236	1·763	1·161	0·198	0·746	0·119	0·243	0·763	0·168
Clec6	1·101	1	1·114	0·067	0·000	0·076	0·104	0·000	0·119
Clec11	1·221	1·436	1·193	0·204	0·342	0·185	0·227	0·437	0·198
Clec37	1·252	1·339	1·240	0·199	0·250	0·192	0·255	0·341	0·243
Clec48	1·022	1·186	1	0·019	0·164	0·000	0·023	0·185	0·000
Clec99	1·268	1·112	1·289	0·267	0·025	0·300	0·273	0·113	0·295
Mean	1·232	1·414	1·207	0·172	0·224	0·164	0·241	0·420	0·215

The allelic richness (Ar), the observed and the expected heterozygosity (Ho and He) are represented between hosts and overall.

**Table 2.** Genetic diversity of the common bed bug over sites with more than 5 individuals

Site	Total sample size	Ar	Ho	He
1	39	1·366	0·211	0·372
2	20	1·462	0·238	0·469
3	18	1·304	0·314	0·304
4	11	1·046	0·064	0·045
5	11	1·065	0·071	0·065
8	6	1·000	0	0
9	30	1·172	0·178	0·172
15	11	1·218	0·104	0·225
19	14	1·444	0·385	0·447
20	21	1·471	0·424	0·472
21	27	1·341	0·298	0·343
23	5	1·044	0	0·057
24	30	1·035	0·035	0·035
25	7	1·277	0·283	0·308
27	39	1·194	0·085	0·196
28	34	1·297	0·113	0·300
30	14	1·164	0·080	0·175

The allelic richness (Ar), the observed and the expected heterozygosity (Ho and He) are represented between hosts and overall.

### Genetic structure

**Figure 2.**
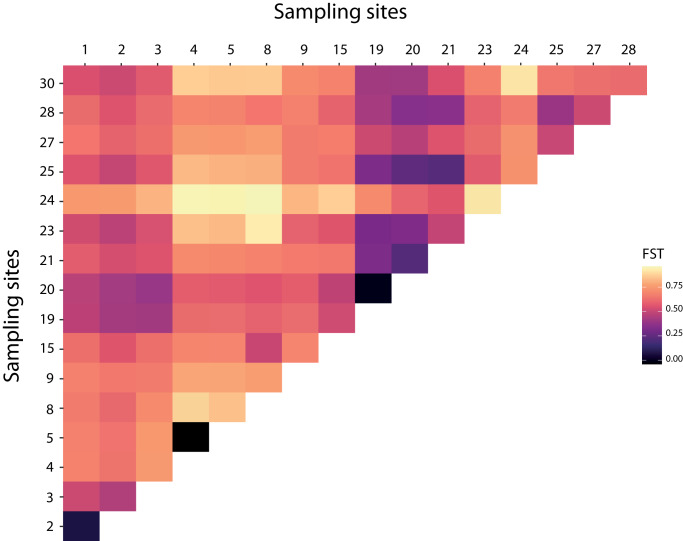
Heatmap of pairwise *F*_ST_ over all pairs of sites based on the microsatellites data.

Over the 10 microsatellite loci, pairwise *F*_ST_ values were estimated to detect genetic structure between and within bat- and human-associated sites. Pairwise *F*_ST_ analyses were consistent with a genetic differentiation among sites (Figure 2 and Table S6). The estimates of the variance components and hierarchical *F*-statistics over all loci were large and significant among sites within host-associated lineages (*F*_SC_ = 0·579, *P* = 0·001); the differentiation between host-associated lineages was also large (*F*_CT_ = 0·254), but not significant when tested using permutations of sites between hosts (*P* = 0·179). However, the 95% confidence interval for *F*_CT_ obtained by bootstrapping over loci did not overlap with 0 (*F*_CT_ 95% CI = [0·008; 0·455]).

**Figure 3.**
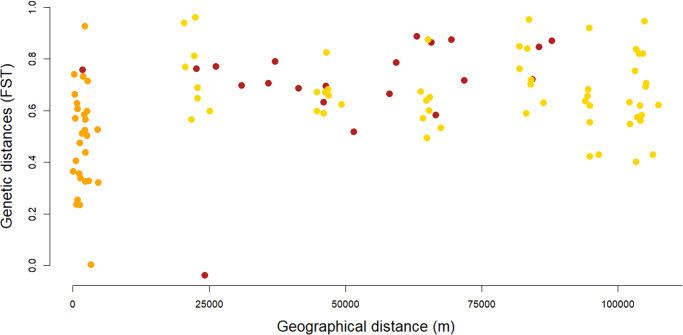
Isolation by distance for human-associated bed bugs. Orange dots highlight pairwise human shelters from the Geneva canton (sites 19–28; Mantel test: *P* = 0·612). Red dots represent pairwise human shelters from outside the Geneva canton (sites 3–15 and 30; Mantel test: *P* = 0·289). Yellow dots show pairwise human shelters from and outside the Geneva canton.

The GLS model highlighted a significant relationship between the host structural matrix and the genetic distances of the bed bugs (GLS model, *t* = −2·987, *p* = 0·003). The bed bugs from the same host showed less genetic differentiation than the samples from the other host. There was also a significant relationship between genetic distances and the structure associated with the Geneva sites (GLS model: *t* = −4·499, *p* < 0·0001). The effect of geographical distances on genetic distances was tested on pairs of bed bugs that belong to Geneva and on pairs of bed bugs from outside Geneva on human-associated bugs only. Both Mantel tests showed non-significant relationships between the geographical and genetic distances (Geneva: *r* = − 0·115, *p* = 0·612; outside Geneva: *r* = 0·087, *p* = 0·289). The gene flow within Geneva or outside Geneva did not depend on the geographical distances (Figure 3).

**Figure 4.**
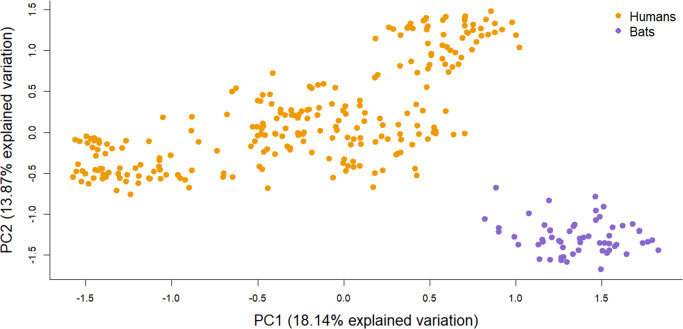
Principal component analysis (PCA) based on the entire microsatellite dataset. After subsampling one individual per sampling site for 100 replicates, 70% of the analysis differentiated the two host-associated bugs into two genetic clusters.

**Figure 5.**
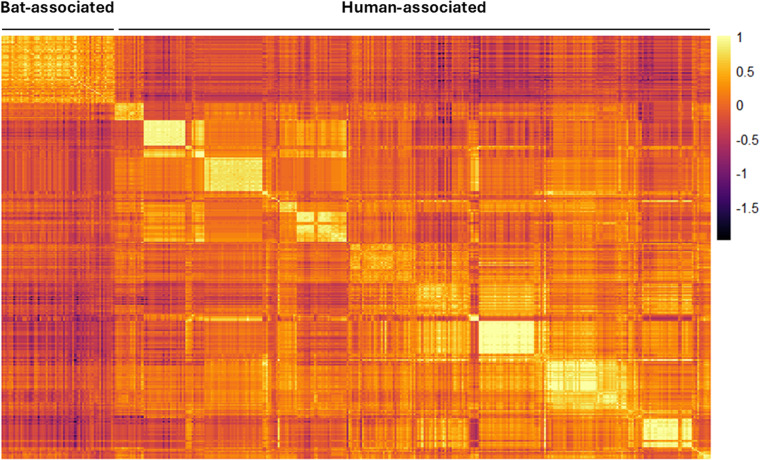
Heatmap of the pairwise kinship over all pairs of individuals based on the microsatellites data.

The PCA analysis revealed a substructure among the different sites and split the individuals by host along the first and second axes (Figure 4). Pairwise kinship between individuals was higher between bugs from the same host and particularly, from the same site for human-associated bugs (Figure 5). The human-associated bugs were closer with bed bugs from the same site whereas the bat-associated bugs showed no substructure.

### Knock-down resistance in the sodium channel

In total, *kdr* genotypes were available for 342 common bed bugs from 25 locations for the V419L, L925I and I936F sites. All 55 bat-associated bugs (from 2 sites) exhibited the wild-type form of the amino acids 419, 925 and 936. In contrast, among the human-associated bed bugs, 1 resistant variant (925I) was present in all but 3 individuals (among 283 mutants out of 286 individuals, 258 were homozygous for the resistant variant and 25 heterozygous), V419 was absent, and I936 was present in a heterozygous state in one individual (Table S7).

## Discussion

Using three types of markers, we showed that bed bugs display the same pattern of genetic differentiation at a very regional scale as at the European scale with two main lineages that are specialized on their respective hosts. The results revealed different structures between human-associated and bat-associated populations. Our results are consistent with previous studies investigating the genetic structure of the two lineages in Europe using mitochondrial, microsatellite markers and *kdr* profiles (Balvín et al., 2012; Booth et al., 2015; Balvín and Booth, 2018). We also showed strong differences between frequencies of resistance alleles as they are only present in the human lineage. Due to the resurgence in human shelters, human-associated bed bugs are heavily exposed to insecticides. This exposure has led to the emergence and increased frequency of a *knock-down resistance* mutation in the voltage-gated sodium channel (Zhu et al., 2010; Dang et al., 2015; Holleman et al., 2019; Lewis et al., 2023). These different selective pressures between bat- and human-associated bugs have led to genetic divergence between the two lineages.

## Host specialization of C. lectularius

Mitochondrial DNA analysis of the COI and 16S concatenated sequences showed a separation between bat- and human-associated bugs (Figure 1A). Unlike previous studies that have found shared haplotypes (Balvín et al., 2012; Booth et al., 2015), the 9 haplotypes associated with humans were not found in the bat-associated bugs. Thus, in this study, bed bugs from the same hosts clustered together. However, when European sequences are included in the analyses, two haplotypes were shared between hosts (Figure 1B; H4 and H5). As it was underlined by Booth et al. (2015), this can be a result of recent introgression, shared alleles, or incorrect assignment of the host. Nevertheless, the comparison between European and local bugs revealed the same pattern of genetic differentiation between larger and smaller scales. Indeed, haplotypes were separated between hosts but not between localities. The bugs from Switzerland displayed the same pattern of genetic differentiation between hosts as the European ones.

The analysis of host differentiation using 10 microsatellite loci was consistent with the mitochondrial haplotype network. Despite the small number of loci, we showed genetic differentiation between the host-associated bugs. PCA revealed two host-associated genetic clusters (Figure 4) indicating the presence of two hosts-specialized lineages suggesting reproductive isolation between bat- and human-associated *C. lectularius*, as it was also demonstrated by Booth et al. (2015). The low level of gene flow between bed bugs from human and bat host species revealed by the pairwise *F*_ST_ analysis is highly consistent with previous population genetics studies in Europe with high overall *F*_ST_ (*F*_ST_ = 0·683, Booth et al., 2015). Despite a large *F*_CT_ (0·254) and a bootstrap CI not overlapping 0, the permutation test for the hierarchical analysis of variance did not show a significant differentiation between the two host-associated lineages. This is most likely due to the lack of power of the permutation test because of the low number of bat roosts (*N* = 2). Despite our sampling and low number of markers, the significant effect of the host structural matrix revealed the strong genetic differentiation between the two host-associated lineages.

Although Wawrocka et al. (2015) have found reproductive isolation between the two lineages from their hybridization experiments, recent studies of cross-experiments suggest otherwise (Devries et al., 2020; Sasínková et al., 2023). The successful reproduction of the two host-associated bed bugs and the presence of reproductive offsprings are revealing the lack of post-copulatory reproductive barriers in the common bed bugs (Devries et al., 2020; Sasínková et al., 2023). Explanations about the maintenance of these genetic differences need further investigations. It is still unclear to what extent bed bugs are able to disperse between their different hosts. Opportunity for host switch depends on the dispersal mechanisms of individuals (Combes, 2004). Contacts between the two hosts are likely to occur allowing the transmission of bed bugs from one host to another. An experimental study has provided evidence that human-associated bed bugs are activated and attracted by human odour and its components (Suchy and Lewis, 2011; Harraca et al., 2012; Liu and Liu, 2015; Devries et al., 2019). Moreover, morphological mechanisms of host detection may be different between host-associated bed bugs (Balvín et al., 2012), but the cues that control these differences are unclear. Heat, exhaled CO_2_ and kairomones are used by other haematophagous insects to detect their host (Lehane, 2005) and could help the bugs to detect and locate their hosts (Rivnay, 1932; Reinhardt and Siva-Jothy, 2007; Suchy and Lewis, 2011). However, there is a lack of information on the host-seeking behaviour of *C. lectularius*, in particular the comparison of host attraction between humans and bats. Balvín et al. (2017) tested pre-mating barriers between the bat- and human-associated bugs by investigating lineage-specific aggregation after blood meals. They found no clear behavioural differences, which means that shelter fidelity does not explain the genetic differentiation between the two lineages. Human-associated bed bugs are more active during the night (Mellanby, 1939; Romero et al., 2010). It may be assumed that the activity period of bat-associated bugs in bat roosts is switched, where bugs would be more active during the day when bats are present, but we found no record of studies investigating this pattern. Understanding the host choice behaviour of the different lineages of the common bed bug could help to understand how it spreads between host species and how the genetic differences between the two lineages are maintained. Moreover, genome-wide association studies could also provide further understanding of the lineages maintenance by identifying specific loci associated with host specialization. Whole genomes of the common bed bugs were previously published (Benoit et al., 2016; Rosenfeld et al., 2016; Miles et al., 2024), providing a valuable resource for future investigations.

### Host-specific population dynamics

Bat-associated bugs showed higher genetic diversity but fewer private alleles than human-associated bugs, possibly due to the low number of bat roosts sampled. The low pairwise *F*_ST_ between the two bat roosts (*F*_ST_ = 0·0769) and the kinship between bat-associated bugs are consistent with a strong gene flow between bugs from these two sites (Figures 2 and 5; Table S6). The two *Myotis myotis* roosts sampled in this study are separated by approximately 17 km, and the estimated population sizes are around 380 and 450 individuals (respectively, sites 1 and 2). *M. myotis* have a low level of genetic differentiation across distant sites in the Alps (separated from 14 to 296 km, Castella et al., (2001)). Therefore, distance between our sampling bat roosts should not act as a barrier to gene flow for both bats and their associated bed bugs. This would be consistent with the low genetic differentiation between the two bat roosts sampled in this study. These results contrast with the dynamic of human-associated bug populations but are consistent with previous studies showing higher nucleotide diversity and allelic richness for bat bugs (Balvín et al., 2012; Booth et al., 2015). Comparison with the European sequences from Booth et al. (2015) allowed to increase the number of sampling sites, along with the number of bat-associated haplotypes. Bat-associated bugs from different sites shared haplotypes, emphasizing the lack of barrier to bugs transmission between bat roosts. Bat- and human-associated sites of bed bugs in Europe could have been colonized independently from foreign sources. Indeed, our results showed a very different picture for human-associated bed bugs, with very limited gene flow. Pairwise *F*_ST_ values were high (Figure 2 and Table S6), and kinships revealed that bugs from the same human shelters were more related than bugs from different sites (Figure 5). Along with a lower genetic diversity, our results show a strong substructure in the human-associated bugs. As small populations tend to be eradicated as quickly as possible, human-associated populations have not had time to grow. Consequently, the low number of multiple infestations (one site exhibiting different haplotypes; Figure S2) and the lower genetic diversity in human-associated bugs suggest that populations were established by founder effects (Figure 1 and Table S5). This supports the theory of a small propagule infestation (Booth et al., 2012, 2018; Saenz et al., 2012). Furthermore, considering that the re-emergence of bed bugs is mainly explained by the increase in transport (Davies et al., 2012; Delaunay, 2012), it is likely that these small founder effects originated from different human sources. To test this hypothesis, it may be of interest to infer the historical demography of the common bed bug in Switzerland. For example, the host-dependent genetic structure of the seabird tick *Ixodes uriae* was explained by the colonization of the northern hemisphere via two independent routes, resulting in two lineages associated with two species of seabirds (Mccoy et al., 2003; Dietrich et al., 2014). In our results, the absence of a relationship between genetic and geographic distances in the human-associated samples, from Geneva and outside Geneva, could be an indication (1) of an absence of gene flow between sites or (2) that the amount of gene flow is not lower between two distant sites compared to two geographically close sites (Booth, 2024). The resolution of the markers could also play a role in the absence of isolation by distance. This result was consistent with other studies (reviewed in Akhoundi et al., 2015; Booth et al., 2018). Significant isolation by distance was found in human-associated bugs in the USA despite a weak relationship that suggests it is more likely that the source populations may come from more distant sites (Saenz et al., 2012; Lewis 2023). Therefore, stepwise colonisation does not explain the observed genetic patterns. It is most likely that bed bugs have colonized human shelters through multiple independent events through human-mediated dispersal. This hypothesis explains the substructure between human sites that are close together. It would be interesting to investigate patterns of isolation by distance between bed bugs on a European scale.

## The resurgence of C. lectularius in human shelters

The resurgence of bed bugs in human shelters has been attributed to insecticide resistance (Romero et al., 2007). Investigation of insecticide resistance in bugs revealed that 99% of the human-associated bed bugs tested had a mutation conferring pyrethroid tolerance (L925I), confirming high exposure to insecticides. This contrasts with the results from bugs collected from bat roosts, where none of the bed bugs sampled have this mutation (all the bat-associated bugs display a wild-type form for the three *kdr* mutations). This resistance to insecticide compounds has already been confirmed in *C. lectularius* with varying intensity depending on the study area (Yoon et al., 2008; Zhu et al., 2010; Booth et al., 2015; Dang et al., 2015; Balvín and Booth, 2018; Lewis et al., 2023). Among the resistant individuals in this study, 8% were heterozygotes (Table S7). Heterozygotes have also been reported in the USA (Lewis et al., 2023). Genotype distribution alone does not provide sufficient information to determine the dominance of the resistance mutation. Further investigations are needed to determine whether heterozygotes exhibit the same level of survival as resistant homozygotes. An experimental survival study exposing the three genotypes (homozygote wild-type, heterozygote, and homozygote mutant) to pyrethroid insecticides would be necessary to assess potential differences in resistance levels. Our results show a high prevalence of the *kdr*-associated resistant human-associated bed bugs in western Switzerland, in agreement with the results of studies in Europe (Durand et al., 2012; Booth et al., 2015; Balvín and Booth, 2018; Vander Pan et al., 2020; Akhoundi et al., 2021). Only one other study investigated the role of the I936F mutation in the pyrethroid resistance of human-associated European bed bugs, and all bat-associated bugs presented the wild-type form of this allele (Balvín and Booth, 2018). Their study found 9 human-associated populations with this mutation including 1 in Switzerland. In agreement with our results and previous studies around the world, the I936F mutation is present at low levels in the human-associated bed bugs (Dang et al., 2015; Palenchar et al., 2015; Lewis et al., 2023). This mutation contributes less to the resistance of pyrethroid insecticides in the bed bugs (Dang et al., 2015).

Additional markers beyond those used in our study have been investigated for detecting insecticide resistance in the common bed bug, from targeted sequencing to transcriptomic analysis and quantitative trait locus mapping (see Dang et al., 2017 for a review). Recently, Haberkorn et al. (2023) have identified a 6 Mb genomic region involved in the pyrethroid resistance in bed bugs. However, it should be acknowledged that this analysis might ignore resistant mutations that are unique to a bed bug population. To further expand the understanding of insecticide resistance, genome-wide association studies could be valuable in identifying mutated genes beyond voltage-gated sodium channel mutations. This approach would provide deeper insight into the extent and underlying mechanisms of resistance in common bed bugs.

*Knock-down resistance* (*kdr*) mutations have been studied to induce pyrethroid tolerance in many animal species (Williamson et al., 1993; Schuler et al., 1998), particularly in other household pests such as several susceptible cockroach species (Liu et al., 2000; Rahimian et al., 2019) or in cat fleas (Bass et al., 2004; Erkunt Alak et al., 2020), highlighting the high frequency of resistance due to the intensive use of pyrethroids. For effective pest control, alternative strategies beyond pyrethroid insecticides must be explored, such as integrated pest management strategies that combine both chemical and non-chemical methods (Karaağaç, 2012).

## Conclusion

Our results are consistent with previous studies that have found host specialization in human and bat bed bugs, and strong genetic structure within human shelters (Balvín et al., 2012; Booth et al., 2012, 2015; Balvín and Booth, 2018). The genetic differences observed among European populations were also present on a smaller, local scale. This is particularly true in this study, where the genetic variations observed locally between the two lineages accurately represented the patterns seen globally. Despite the absence of reproductive barriers (Devries et al., 2020; Sasínková et al., 2023), the limited gene flow between the lineages, explained by the morphological differences (Balvín et al., 2012) and the potential different host seeking behaviour, has resulted in increased host fidelity and specialization. Furthermore, the introduction of small propagules from multiple sources and the quick eradication of bugs in human shelters have resulted in a strong genetic structure and insecticide resistance in the human lineage.

## Supporting information

Castex et al. supplementary material Castex et al. supplementary material

## Data Availability

All data used in the analysis will be deposited in Mendeley Data.

## Supplementary material

The supplementary material for this article can be found at https://doi.org/10.1017/S0031182025000575.
