# Supplementary material for: Genetic characterization of the bat and human lineages of the common bed bug (Cimex lectularius) at a local scale
Source: Parasitology. 2025 Apr 21;152(5):510–21. doi: 10.1017/S0031182025000575 (PMC12278015; doi:10.1017/S0031182025000575)
Supplement: Castex et al. supplementary material [file S0031182025000575sup001.docx]

**Supplementary Figures & Tables**


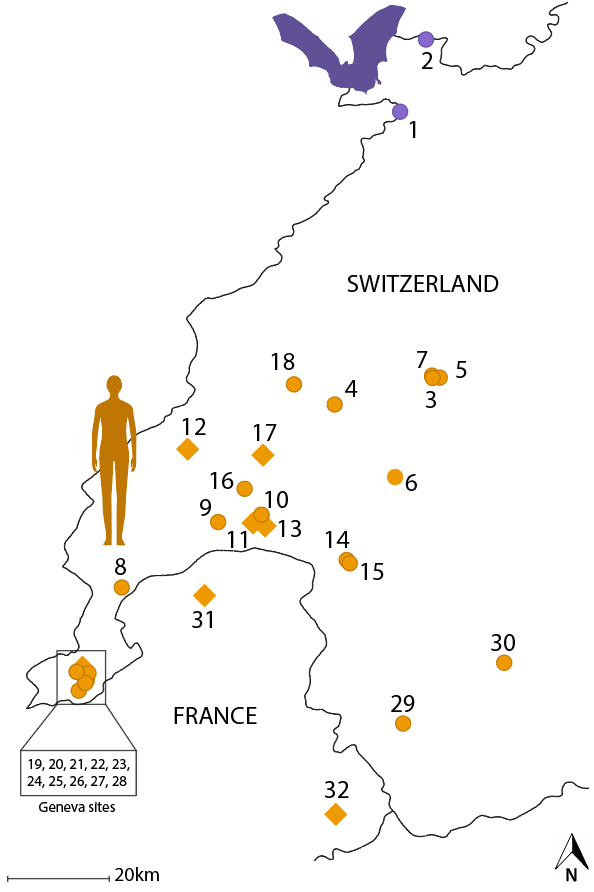


**Figure S1.** Map of the 32 sites used in the study. Diamonds means the coordinates are approximated because they were unavailable. The bat roosts are in green, and the human shelters are in purple. The sites 19 to 28 are all located in the Geneva canton.

**Table S1.** Number of individuals from the 32 sites used in the different analyses. In the host column, B stands for bat and H for human.

|  |  |  | Analysis | | |
| --- | --- | --- | --- | --- | --- |
| Host | Site | Total sample size | Mitochondrial | Microsatellites | *Kdr* haplotypes |
| B | 1 | 39 | 32 | 39 | 38 |
| B | 2 | 20 | 20 | 20 | 17 |
| H | 3 | 18 | 14 | 15 | 15 |
| H | 4 | 11 | 7 | 11 | 11 |
| H | 5 | 11 | 10 | 11 | 11 |
| H | 6 | 3 | 3 | 3 | 3 |
| H | 7 | 2 | 0 | 2 | 2 |
| H | 8 | 6 | 0 | 5 | 5 |
| H | 9 | 30 | 0 | 30 | 30 |
| H | 10 | 3 | 3 | 3 | 2 |
| H | 11 | 10 | 2 | 6 | 5 |
| H | 12 | 3 | 0 | 0 | 0 |
| H | 13 | 13 | 10 | 0 | 0 |
| H | 14 | 24 | 0 | 24 | 24 |
| H | 15 | 11 | 0 | 11 | 11 |
| H | 16 | 1 | 0 | 1 | 1 |
| H | 17 | 1 | 1 | 0 | 0 |
| H | 18 | 1 | 1 | 1 | 1 |
| H | 19 | 14 | 12 | 14 | 14 |
| H | 20 | 21 | 20 | 21 | 21 |
| H | 21 | 27 | 23 | 26 | 26 |
| H | 22 | 1 | 1 | 1 | 0 |
| H | 23 | 5 | 4 | 5 | 4 |
| H | 24 | 30 | 25 | 29 | 29 |
| H | 25 | 7 | 4 | 7 | 2 |
| H | 26 | 4 | 3 | 3 | 1 |
| H | 27 | 39 | 25 | 38 | 38 |
| H | 28 | 34 | 29 | 37 | 25 |
| H | 29 | 1 | 1 | 1 | 0 |
| H | 30 | 6 | 6 | 6 | 6 |
| H | 31 | 5 | 5 | 0 | 0 |
| H | 32 | 14 | 2 | 0 | 0 |
|  | TOTAL SITES |  | 25 | 27 | 25 |
|  | TOTAL INDIVIDUALS |  | 263 | 370 | 342 |

**Table S2.** P-values of genotypic disequilibrium per pair of loci. Adjusted p-value for 5% nominal level is 0.0011. Genotypic disequilibrium was estimated with Delta’.

|  | BB21B | BB29B | BB31B | BB38B | BB42B | Clec6 | Clec11 | Clec37 | Clec48 | Clec99 |
| --- | --- | --- | --- | --- | --- | --- | --- | --- | --- | --- |
| BB21B | 0 |  |  |  |  |  |  |  |  |  |
| BB29B | 0.152 | 0 |  |  |  |  |  |  |  |  |
| BB31B | 0.523 | 0.474 | 0 |  |  |  |  |  |  |  |
| BB38B | 0.564 | 0.114 | 0.923 | 0 |  |  |  |  |  |  |
| BB42B | 0.283 | 0.137 | 0.130 | 0.961 | 0 |  |  |  |  |  |
| Clec6 | NA | NA | NA | NA | NA | 0 |  |  |  |  |
| Clec11 | 0.848 | 0.783 | 0.296 | 0.921 | 0.432 | NA | 0 |  |  |  |
| Clec37 | 0.010 | 0.038 | 0.719 | 0.254 | 0.391 | NA | 0.704 | 0 |  |  |
| Clec48 | 0.559 | 0.797 | 0.852 | 0.431 | 0.363 | NA | 0.641 | 0.683 | 0 |  |
| Clec99 | 0.212 | 0.624 | 0.098 | 0.112 | 0.728 | NA | 0.508 | 0.788 | 1.000 | 0 |

**Table S3.** Collinearity of the structural matrices. The correlations between the different structural matrices were tested using mantel tests. xdis and ydis are the different distance matrices tested in the mantel test. Host_matrix represent the structure between pairs of sites coming from the same host (bat lineage/bat lineage or human lineage/human lineage; encoded as 1) or not (bat lineage/human lineage; encoded as 0). GE_matrix represent the structure between pairs of sites coming from the same region (Geneva site/Geneva site or outside Geneva site/outside Geneva site; encoded as 1) or not (Geneva site/outside Geneva site; encoded as 0). Geodist_matrix represent the geographic distances between pairs of sites. r is the mantel statistic, representing the correlation between the matrices and *p*, the associated p-value.

| xdis | ydis | r | *p* |
| --- | --- | --- | --- |
| - Host_matrix | Geodist_matrix | 0.6562 | 0.012 |
| - GE_matrix | Geodist_matrix | 0.5267 | 0.001 |
| Host_matrix | GE_matrix | 0.0042 | 0.472 |

**Table S4.** Distribution of haplotypes and number of associated individuals per site.

| Sites | Host | Haplotypes (nb of individuals) |
| --- | --- | --- |
| 1 | Bat | H10 (32) |
| 2 | Bat | H10 (19), H11 (1) |
| 3 | Human | H1 (13), H2 (1) |
| 4 | Human | H3 (7) |
| 5 | Human | H3 (10) |
| 6 | Human | H6 (3) |
| 10 | Human | H3 (3) |
| 11 | Human | H3 (1), H7(1) |
| 13 | Human | H5 (4), H8 (6) |
| 14 | Human | H3 (1), H4 (2) |
| 17 | Human | H8 (1) |
| 18 | Human | H1 (1) |
| 19 | Human | H3 (12) |
| 20 | Human | H3 (20) |
| 21 | Human | H3 (23) |
| 22 | Human | H4 (1) |
| 23 | Human | H5 (5) |
| 24 | Human | H6 (25) |
| 25 | Human | H6 (4) |
| 26 | Human | H1 (3) |
| 27 | Human | H3 (25) |
| 28 | Human | H1 (8), H2(1), H3(20) |
| 29 | Human | H8 (1) |
| 30 | Human | H3 (6) |
| 31 | Human | H9 (5) |
| 32 | Human | H1 (2) |

**
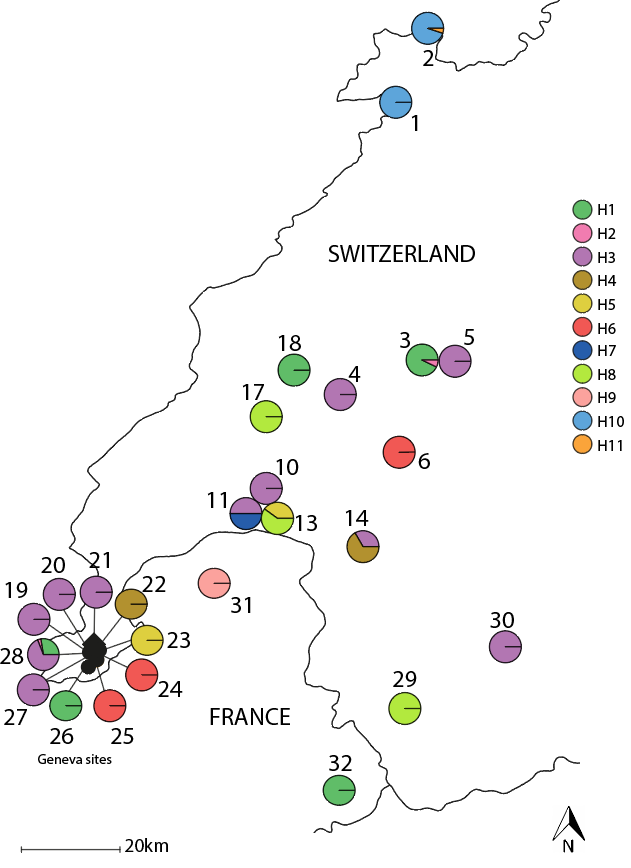
**

**Figure S2.** Distribution of the different haplotypes per sites with proportion of individuals per haplotypes.

**Table S5.** Rarefied allelic count per locus and sampling site. The first two sites are bat-associated.

|  | **1** | **2** | **3** | **4** | **5** | **8** | **9** | **15** | **19** | **20** | **21** | **23** | **24** | **25** | **27** | **28** | **30** |
| --- | --- | --- | --- | --- | --- | --- | --- | --- | --- | --- | --- | --- | --- | --- | --- | --- | --- |
| **BB21B** | 1.673 | 1.718 | 1.545 | 1 | 1.167 | 1 | 1.066 | 1.325 | 1.464 | 1.672 | 1.075 | 1.356 | 1 | 1 | 1.084 | 1.054 | 1.356 |
| **BB29B** | 1.474 | 1.570 | 1.515 | 1 | 1 | 1 | 1 | 1.623 | 1.680 | 1.685 | 1.672 | 1 | 1 | 1 | 1.609 | 1.355 | 1 |
| **BB31B** | 1.440 | 1.527 | 1.500 | 1 | 1 | 1 | 1 | 1 | 1.700 | 1.633 | 1.568 | NA | 1 | 1.733 | 1 | 1.149 | 1.356 |
| **BB38B** | 1.534 | 1.676 | 1.405 | 1.455 | 1.485 | 1 | 1.341 | 1 | 1.508 | 1.444 | 1.340 | 1 | 1 | 1.495 | 1.425 | 1.576 | 1.200 |
| **BB42B** | 1.734 | 1.792 | 1.077 | 1 | 1 | 1 | 1.756 | 1.479 | 1 | 1 | 1.212 | NA | 1 | 1 | 1.274 | 1.456 | 1 |
| **Clec6** | 1 | 1 | 1 | 1 | 1 | 1 | 1 | 1 | 1.271 | 1.438 | 1.177 | 1 | 1 | 1.485 | 1 | 1.344 | 1 |
| **Clec11** | 1.421 | 1.450 | 1.480 | 1 | 1 | 1 | 1 | 1 | 1.476 | 1.470 | 1.483 | 1 | 1 | 1 | 1.548 | 1.435 | 1 |
| **Clec37** | 1.312 | 1.367 | 1 | 1 | 1 | 1 | 1.066 | 1.523 | 1.566 | 1.610 | 1.398 | 1 | 1 | 1.409 | 1 | 1.496 | 1.533 |
| **Clec48** | 1.075 | 1.296 | 1 | 1 | 1 | 1 | 1 | 1 | 1 | 1 | 1 | 1 | 1 | 1 | 1 | 1 | 1 |
| **Clec99** | 1 | 1.224 | 1.519 | 1 | 1 | 1 | 1.494 | 1 | 1.775 | 1.758 | 1.487 | 1 | 1.349 | 1.648 | 1 | 1.105 | 1.200 |

**Table S6.** Estimation of *C. lectularius* genetic differentiation between bat and human-associated population from 17 locations in Western Switzerland represented by pairwise F_ST_, after Bonferroni correction.

|  | **1** | **2** | **3** | **4** | **5** | **8** | **9** | **15** | **19** | **20** | **21** | **23** | **24** | **25** | **27** | **28** |
| --- | --- | --- | --- | --- | --- | --- | --- | --- | --- | --- | --- | --- | --- | --- | --- | --- |
| **2** | 0.0769 |  |  |  |  |  |  |  |  |  |  |  |  |  |  |  |
| **3** | 0.5235 | 0.4637 |  |  |  |  |  |  |  |  |  |  |  |  |  |  |
| **4** | 0.6865 | 0.6434 | 0.7606 |  |  |  |  |  |  |  |  |  |  |  |  |  |
| **5** | 0.6822 | 0.6392 | 0.7571 | -0.0380 |  |  |  |  |  |  |  |  |  |  |  |  |
| **8** | 0.6644 | 0.6134 | 0.7197 | 0.8858 | 0.8464 |  |  |  |  |  |  |  |  |  |  |  |
| **9** | 0.6808***** | 0.6520***** | 0.6643***** | 0.7899***** | 0.7848***** | 0.7705***** |  |  |  |  |  |  |  |  |  |  |
| **15** | 0.6326 | 0.5688 | 0.6314 | 0.7048 | 0.6949 | 0.5175 | 0.6973***** |  |  |  |  |  |  |  |  |  |
| **19** | 0.4881 | 0.4350 | 0.4279 | 0.6290 | 0.6218 | 0.5971 | 0.6220***** | 0.5320 |  |  |  |  |  |  |  |  |
| **20** | 0.4819 | 0.4293***** | 0.4010 | 0.5881***** | 0.5824 | 0.5646 | 0.5883***** | 0.4923***** | 0.0020 |  |  |  |  |  |  |  |
| **21** | 0.5900 | 0.5462***** | 0.5608 | 0.7175 | 0.7062 | 0.6883 | 0.6585***** | 0.6505 | 0.3251 | 0.2336 |  |  |  |  |  |  |
| **23** | 0.5356 | 0.4850 | 0.5484 | 0.8482 | 0.8359 | 0.9375 | 0.5981 | 0.5697 | 0.3211 | 0.3370 | 0.5013 |  |  |  |  |  |
| **24** | 0.7592***** | 0.7624***** | 0.8206 | 0.9514 | 0.9452 | 0.9611 | 0.8250***** | 0.8746***** | 0.7128***** | 0.6056***** | 0.5683***** | 0.9248 |  |  |  |  |
| **25** | 0.5638 | 0.5062 | 0.5744 | 0.8384 | 0.8196 | 0.8113 | 0.6698 | 0.6378 | 0.3276 | 0.2544 | 0.2356 | 0.5838 | 0.7397 |  |  |  |
| **27** | 0.6460 | 0.6018***** | 0.6312 | 0.7608 | 0.7525 | 0.7673***** | 0.6703***** | 0.6733***** | 0.5259***** | 0.4747***** | 0.5637***** | 0.6276 | 0.7316***** | 0.5111 |  |  |
| **28** | 0.6286 | 0.5608 | 0.6193 | 0.7009 | 0.6912 | 0.6463 | 0.6811***** | 0.5995***** | 0.4379***** | 0.3544***** | 0.3630***** | 0.5966 | 0.6613***** | 0.4046 | 0.5227***** |  |
| **30** | 0.5521 | 0.5243 | 0.5816 | 0.8737 | 0.8624 | 0.8693 | 0.7167***** | 0.6867 | 0.4279 | 0.4223 | 0.5548 | 0.6806 | 0.9183***** | 0.6559 | 0.6364***** | 0.6181 |

*****Statistically significant.

**Table S7.** Sample information for the knock-down resistance mutation on amino acid 419, 925 and 936 for *C. lectularius*, using the annotation of Lewis et al. (2023). wt: wild-type (absence of mutation in the three amino acids: V419, L925 and I936), L925I: mutant for the amino acid 925 (leucine replaced by isoleucine), L925I*^het^*: mutant heterozygote for the amino acid 925, I936F: mutant for the amino acid 936 (isoleucine replaced by phenylalanine) and I936F*^het^*: mutant heterozygote for the amino acid 936. The bat-associated sites are 1 and 2.

| Site | Sample Size | Host species | wt | L925I | L925I*^het^* | I936F | I936F*^het^* |
| --- | --- | --- | --- | --- | --- | --- | --- |
| 1 | 38 | *Myotis myotis* | 38 | 0 | 0 | 0 | 0 |
| 2 | 17 | *Myotis myotis* | 17 | 0 | 0 | 0 | 0 |
| 3 | 15 | *Homo sapiens* | 0 | 15 | 0 | 0 | 0 |
| 4 | 11 | *Homo sapiens* | 0 | 11 | 0 | 0 | 0 |
| 5 | 11 | *Homo sapiens* | 0 | 11 | 0 | 0 | 0 |
| 6 | 3 | *Homo sapiens* | 0 | 3 | 0 | 0 | 0 |
| 7 | 2 | *Homo sapiens* | 0 | 2 | 0 | 0 | 0 |
| 8 | 5 | *Homo sapiens* | 0 | 5 | 0 | 0 | 0 |
| 9 | 30 | *Homo sapiens* | 0 | 30 | 0 | 0 | 0 |
| 10 | 2 | *Homo sapiens* | 0 | 1 | 1 | 0 | 0 |
| 11 | 5 | *Homo sapiens* | 0 | 1 | 4 | 0 | 0 |
| 14 | 24 | *Homo sapiens* | 2 | 22 | 0 | 0 | 0 |
| 15 | 11 | *Homo sapiens* | 0 | 11 | 0 | 0 | 0 |
| 16 | 1 | *Homo sapiens* | 0 | 0 | 0 | 0 | 1 |
| 18 | 1 | *Homo sapiens* | 0 | 1 | 0 | 0 | 0 |
| 19 | 14 | *Homo sapiens* | 0 | 14 | 0 | 0 | 0 |
| 20 | 21 | *Homo sapiens* | 0 | 21 | 0 | 0 | 0 |
| 21 | 26 | *Homo sapiens* | 0 | 26 | 0 | 0 | 0 |
| 23 | 4 | *Homo sapiens* | 0 | 4 | 0 | 0 | 0 |
| 24 | 29 | *Homo sapiens* | 0 | 29 | 0 | 0 | 0 |
| 25 | 2 | *Homo sapiens* | 0 | 1 | 1 | 0 | 0 |
| 26 | 1 | *Homo sapiens* | 0 | 0 | 1 | 0 | 0 |
| 27 | 38 | *Homo sapiens* | 0 | 31 | 7 | 0 | 0 |
| 28 | 24 | *Homo sapiens* | 0 | 13 | 11 | 0 | 0 |
| 30 | 6 | *Homo sapiens* | 0 | 6 | 0 | 0 | 0 |
